# Health promotion in German kindergartens—design and methods of the exploratory, cluster-randomized, mixed methods KNEIPP–KITA Bavaria study

**DOI:** 10.3389/fmed.2025.1585322

**Published:** 2025-05-29

**Authors:** Miriam Ortiz, Christine Bernardi, Christine Welker, Lisa Boyer, Julia von Sommoggy, Hansjoerg Baurecht, Stephanie Roll, Tatjana Tissen-Diabaté, Anne Herrmann, Benno Brinkhaus

**Affiliations:** ^1^Institute of Social Medicine, Epidemiology and Health Economics, Charité - Universitätsmedizin Berlin, Corporate Member of Freie Universität Berlin and Humboldt-Universität zu Berlin, Berlin, Germany; ^2^Medical Sociology, Department of Epidemiology and Preventive Medicine, University of Regensburg, Regensburg, Germany; ^3^Department of Epidemiology and Preventive Medicine, University of Regensburg, Regensburg, Germany; ^4^Institute of Social Medicine and Health System Research, Medical Faculty University Hospital Magdeburg, Magdeburg, Germany; ^5^Department of Hematology and Oncology, University Hospital Regensburg, Regensburg, Germany; ^6^Bavarian Cancer Research Center (BZKF), Erlangen, Germany

**Keywords:** kindergarten, health prevention, Kneipp Health Concept, health promotion, hydrotherapy

## Abstract

**Background:**

The Kneipp Health Concept, which can be traced back to the German Sebastian Kneipp, integrates five health-promoting elements: physical activity, healthy nutrition, medicinal herbs, mental wellbeing and hydrotherapy. This concept is embedded in a pedagogical framework for children and has been adopted by more than 500 kindergartens across Germany and in other countries. The KNEIPP–KITA study Bavaria aims to evaluate the short- and medium-term effects of KHC on the health and wellbeing of children, their parents, and educational staff.

**Methods:**

This explorative, cluster-randomized, matched-pair, two-arm controlled, mixed-methods study is being conducted in 10 kindergartens, which are randomly assigned to either the Kneipp group (implementing the Kneipp Health Concept as intervention) or a control group. Children aged 3 years and older, their parents, and kindergarten staff are included in the study, with a follow-up period of 24 months. Staff in the Kneipp group receive a specific training in the five elements of Kneipp Health Concept, after which they integrate these components into daily routine of their kindergarten. The control group continues their usual activities without implementing the Kneipp Health Concept. Outcome parameters (assessed through questionnaires in children, parents and staff) include health status, health consciousness, health-related behavior, family health, and quality of life at 9, 15, and 21 months after the Kneipp Health Concept training (start of intervention). In addition, barriers and facilitators of the implementation are explored in a qualitative sub-study through semi-structured interviews conducted with kindergarten staff and parents in the Kneipp group at baseline and 14–16 months post-training. Data analyses will be carried out at both the individual and cluster level, with separate analyses for children, parents, and staff. The qualitative data will be analyzed using the Framework Method following the approach outlined by Gale et al.

**Discussion:**

The results of the study pave the way for a sustainable implementation of the Kneipp Health Concept. In addition, this exploratory study will inform the planning of future intervention studies with valuable data material and contribute to the development and evaluation of effective childhood prevention strategies.

**Trial registration:**

Deutsches Register Klinischer Studien DRKS (German clinical trials register) ID: DRKS00031865.

## 1 Introduction

The health status of children and adolescents in Germany remains a key focus of ongoing research due to the important role of childhood health in the prevention of non-communicable diseases like obesity and metabolic syndrome later in life (1). The body mass index as an outcome influenced by lifestyle habits and socioeconomic status is a common indicator of health including health related quality of life (2). Obesity in childhood is associated with a higher risk to develop obesity in adulthood adults (3). Around 15.4% of children and adolescents at the age of 3–17 years in Germany are overweight, with 6% classified as obese (4). Among preschool children aged 3–6 years, the prevalence of overweight is 10.8% for girls and 7.3% for boys. This data from the second wave of the German KiGGS study on children and adolescents (2014–2017), conducted by the Robert Koch Institute, indicate that the rates of overweight have remained overall stable and declined in children at age 3–6 years since the first survey wave in 2003–2006 (5). This may be attributed to the effectiveness of existing preventive measures. The national health goal “Growing up healthy: life skills, health and nutrition”, first formulated by the German government in 2003 and updated several times, underscores the importance of the preventive measures. Achieving this goal requires health promotion and prevention efforts to begin in early childhood, such as in (specially trained) kindergartens (6).

Setting-based approaches to prevention have been utilized in Germany for many years, particularly in promoting health through increased physical activity and healthy nutrition in kindergartens. These preventive measures aim, among other objectives, to foster equal opportunities for good health, given social inequalities in Germany (7). Although multiple factors influence children's health, several studies have highlighted the role of kindergartens in preventing issues like obesity (8). However, the broader impact of caregivers and childcare environments on children's overall health remains uncertain (9). The present study aims to address these gaps by exploring a specific concept related to health promotion in kindergarten settings.

The Kneipp Health Concept for kindergartens is rooted in the health philosophy of Sebastian Kneipp (1821–1897), a German priest and non-medical health care practitioner who was a leading figure in traditional European medicine, particularly in German-speaking countries (10). The Kneipp Health Concept integrates components of healthy nutrition and physical activity with hydrotherapy, mental wellbeing (referred to as “order of life”), and medicinal herbs and plants (11). This holistic approach provides comprehensive health education and promotes wellbeing through natural elements. The Kneipp Health Concept for kindergartens has been developed for this setting and refined over the past 20 years by the Kneipp Association, a large German lay organization for nature based preventive measures according to S. Kneipp (12, 13). By 2024, over 500 kindergartens in Germany and 80 kindergartens in Austria have been certified by the Kneipp Association after receiving specialized Kneipp Health Concept training and successfully implementing the program. The concept is currently implemented as well in Lithuania and South Korea. While there have been no comprehensive scientific evaluations of the effects of Kneipp Health Concept on children‘s health, two pilot studies have reported promising initial results. These studies indicate that hydrotherapy interventions helped reducing respiratory tract infections and absenteeism among children in Kneipp kindergartens (14, 15). Furthermore, the feedback from the pedagogical staff at Kneipp kindergartens suggests significant improvements in various aspects of children's health due to the implementation of preventive measures. Educators themselves have reported improved health and increased motivation at work (16). Given these positive initial findings, a more comprehensive scientific evaluation of the Kneipp Health Concept, in a methodologically robust study focusing on health-related outcomes is crucial to fully understanding its impact and efficacy.

### 1.1 Objectives

The aim of this exploratory study is to evaluate the potential effectiveness, feasibility and safety under real life conditions of the Kneipp Health Concept in kindergartens for children, their parents, and pedagogical staff, with a particular focus on health outcomes such as upper respiratory tract infections, the adoption of health-promoting lifestyle habits, and quality of life.

## 2 Methods

This study is a collaboration between the University of Regensburg and the Charité - Universitätsmedizin Berlin, Germany. We are conducting the study in kindergartens in Bavaria, in particular in the city of Regensburg and in the greater Regensburg area.

The study protocol (Vs. 20230228) received approval from the Ethics Committee of the University of Regensburg in March 2023 (approval number 23-3298-101).

### 2.1 Design

The *KNEIPP–KITA study Bavaria* is a two-arm, cluster-randomized, controlled, parallel-group, open, exploratory clinical trial utilizing mixed-methods. The study is characterized by a pragmatic approach (17, 18) through the real world setting in a kindergarten and participants that are part of this setting: children, their parents and pedagogical staff, and an intervention, that may affect the setting itself and the participants. For pragmatic reasons we decided for a waitlist control.

**Figure 1:**
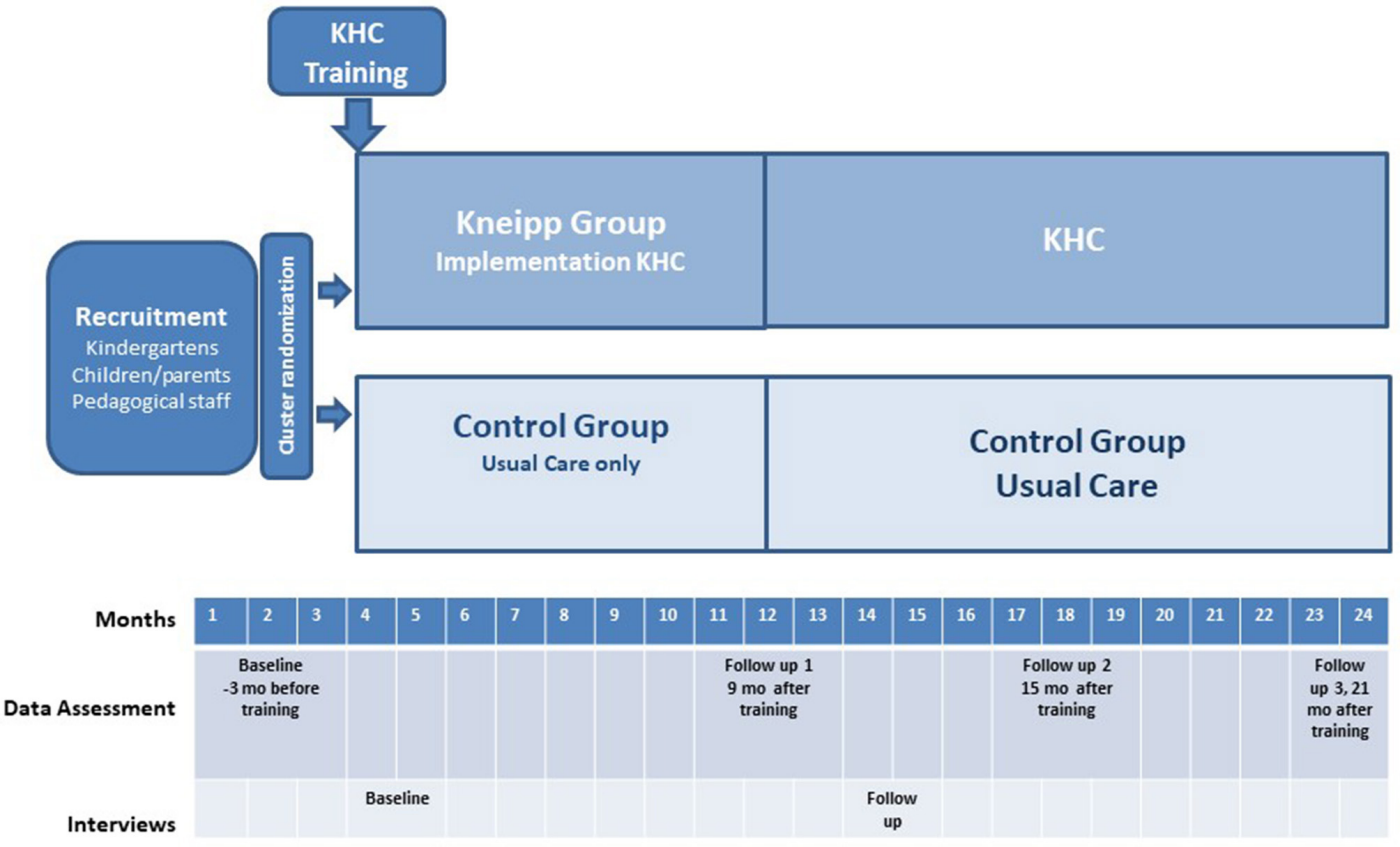
Study design.

The study started with a 3-month recruitment phase, during which kindergartens along with children, parents, and pedagogical staff were enrolled. The kindergartens (cluster) were then randomized into two matched groups: the Kneipp group, which implements the Kneipp Health Concept after completing specialized training, or the control group, which continues their usual practices without including it. The study's total duration is 24 months, with an individual observation period ranging from 21 to 24 months, depending on the timing of each baseline assessment (Figure 1).

### 2.2 Recruitment of kindergartens and participants

Kindergarten recruitment began in March 2023, targeting all kindergartens within a 200 km radius of Regensburg. Eligible kindergartens were pre-screened and contacted by telephone to gauge interest in participation. Study details were also shared via the Kneipp Association to ensure sufficient enrollment. Once a kindergarten agreed to participate, recruitment of children, parents and pedagogical staff commenced and was completed prior to the kindergarten's random allocation.

Following consultation with their staff, kindergarten heads agreed to participate in the study by signing a written commitment in the form of a contract detailing research-related activities. They had received thorough written and oral information about the study beforehand. Participants' recruitment took place from May to September 2023, data collection is expected to conclude in October 2025. Written informed consent was obtained from parents and kindergarten staff which also involved asking for permission to use their data if they withdraw.

### 2.3 Participant and public involvement

Stakeholders, including parents, pedagogical staff, kindergarten administrators, were actively involved in the preparation of the study to ensure that the data collection processes in both the kindergartens and family settings were feasible. They helped to refine study documents, such as questionnaires and diaries.

### 2.4 Eligibility criteria

#### 2.4.1 Kindergartens

The inclusion criteria for the kindergartens were: (I) interest in implementing the Kneipp Health Concept including the staff training; (II) willingness to be randomly assigned to one of two study groups; (III) if placed in the control group, a commitment not to implement the KHC for the next 24 months; (IV) kindergartens needed to have at least 40 childcare places. Exclusion criteria included: Kindergartens with specific ideological or educational approaches, such as Waldorf or Montessori Kindergartens.

#### 2.4.2 Children, parents, and pedagogical staff

The study includes children in kindergartens aged three and older, with parental consent if they were expected to remain in the kindergarten for further 2 years. One parent per participating child who was at least 18 years old, lived in the same household as the child and who was willing to complete the questionnaires, was included.

Pedagogical staff of legal age, motivated to be trained and work with the Kneipp Health Concept (if their kindergarten was assigned to the Kneipp group) and expected to remain in the kindergarten for at least 2 years, were also eligible. Children, parents, or staff with serious instable mental or physical health conditions were excluded. On the personal level, participants are allowed to discontinue the study without giving reasons at any time.

### 2.5 Study intervention

The study intervention involves implementing the Kneipp Health Concept by integrating it into daily kindergarten routines following the training of the pedagogical staff. In practice, various Kneipp Health Concept activities can be tailored to the children's needs and the kindergarten's specific conditions. However, to ensure a semi-standardized approach, the following health-promoting elements, all integral to the Kneipp Health Concept, training will be implemented in the participating kindergartens:

- Hydrotherapy: Water-based procedures such as warm or cold arm or foot baths, water pouring, or dew/water walking, performed at least 4–5 times per week.
- Mental wellbeing: Incorporating daily rituals, such as morning circle, along with regular phases of activation and relaxation. Activities, conducted 1–2 times per week, may include mindfulness exercise, stillness practices, massages and relaxation therapies.
- Physical activity: Engaging in physical activity at least 3 times per week and exercise for a minimum of 30 min, including outdoor physical activity (at least twice a week, weather permitting) and group physical activity therapy, such as gymnastics (at least once a week).
- Healthy nutrition: Following the nutrition guidelines of the German Association for Food for kindergartens and teaching the principles of a healthy diet at least once a week (19).
- Medicinal herbs: Introducing and teaching the basics of local medicinal herbs and plants, maintaining herb gardens, and involving children in activities such as making oils, teas, tinctures, ointments, or bath salts, at least once a week.

**Figure 2:**
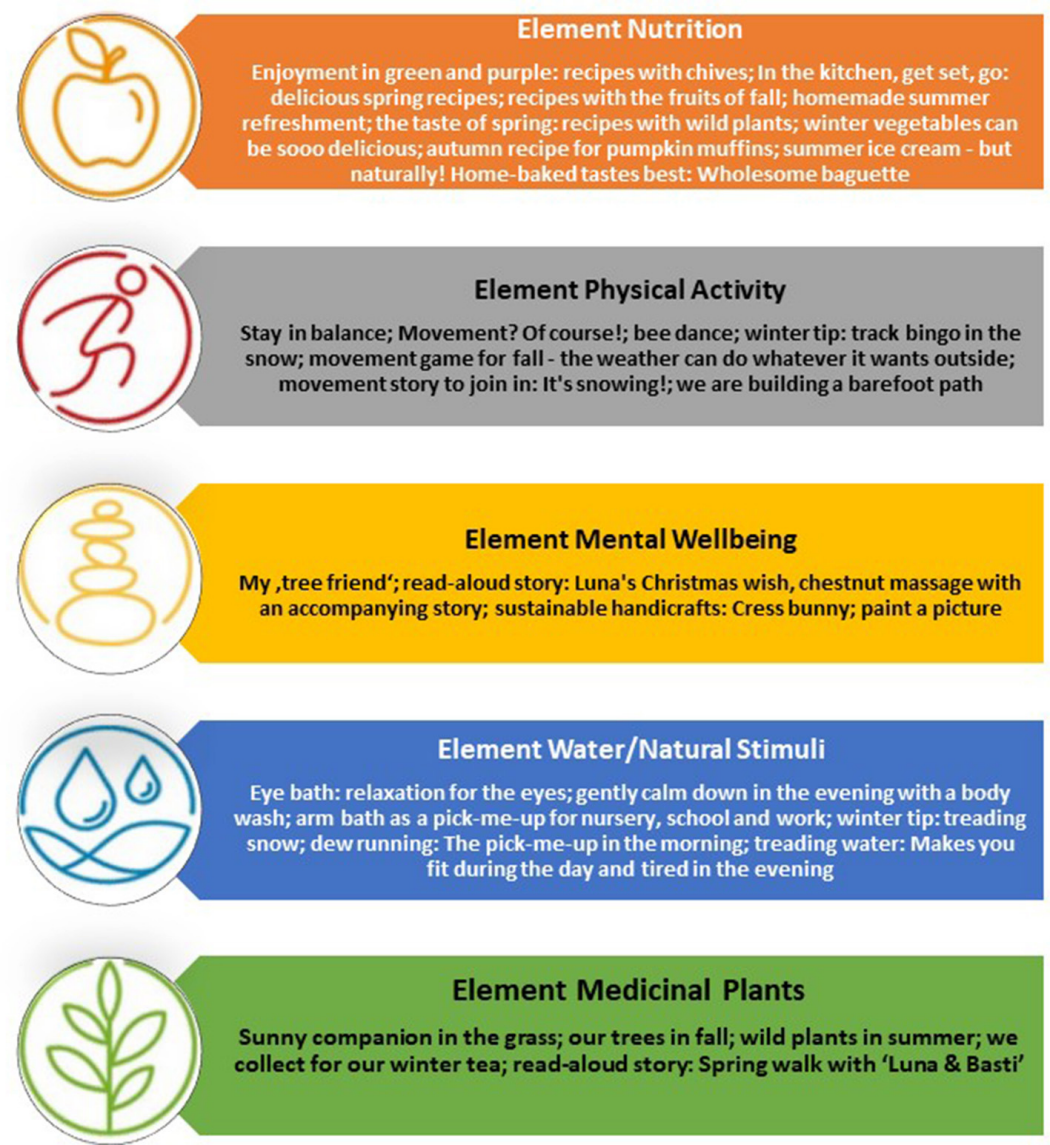
Elements of the Kneipp Health Concept. Icons reproduced with permission from Kneipp Bund e.V.

The Kneipp Association website provides explanations and videos about the Kneipp health preventive elements which can be used by study participants free of charge in both German and English, with an example (Figure 2) (13). Parents of children in kindergartens can be addressed through informational materials at parents' meetings, and practical activities related to Kneipp treatments. During the 2-years study, the control group is prohibited from implementing the Kneipp Health Concept, but will have the opportunity to receive a training after the study termination.

To support recruitment and adherence, participating kindergartens receive financial compensation for Kneipp Health Concept training and implementation expenses (e.g., bathtubs), in the Kneipp group after enrollment and in the control group after the end of the study. Furthermore, participating pedagogical staff and parents will receive their individual study results, and parents will also receive an incentive of 30 Euros upon study completion. Throughout the study, participants are regularly contacted to ensure adherence and receive newsletters with encouragement.

### 2.6 Instruments and outcomes

Since this is an exploratory trial, no primary outcome was predefined. We hypothesize the Kneipp Health Concept to influence three main health-related topics in children and Kindergarten staff: (1) the number and severity of upper respiratory tract infections, (2) lifestyle changes and (3) Health-related quality of life. We consider these areas to be equally relevant outcomes and have therefore chosen not to prioritize outcomes.

Outcome data will be assessed at 9, 15, and 21 months after beginning of intervention. Due to the staggered kindergarten recruitment, baseline data were collected within a 3 months window before the Kneipp Health Concept training commenced. The three follow-up assessments were aligned with the Kneipp Health Concept training dates for the Kneipp group, ensuring parallel assessment for matched pairs. Figure 1 provides the design and an overview of the assessment timepoints with details in Supplementary Table 1.

#### 2.6.1 Children

Children's health status is tracked by parents in questionnaires and weekly diaries, including height, weight (in kg and percentiles); BMI; health resources utilized; number of days absent; occurrence of chronic diseases and respiratory tract infections; number of nights in inpatient treatment; weekly physical activity; time spent outdoors; nutrition per week; screen time.

Physical and mental wellbeing are assessed using a selection of questions from the KINDL-R questionnaire (20, 21).

#### 2.6.2 Parents

The following data and outcomes are collected: Individual health consciousness using the Health Consciousness Scale-German (HCS-G) (22, 23); health-related quality of life through Short Form-12 (SF-12) (24, 25); use of TCIM (traditional, complementary and integrative medicine) based on selected questions from the I-CAM-G (26); family climate using the modified family climate scale by Schneewind et al. as utilized in the KIGGS study (27, 28), and the frequency of family meals; health behaviors/lifestyle habits including physical activity and sedentary behavior from the Global Physical Activity Questionnaire (GPAQ) (29, 30); psychosocial stress using the Distress Thermometer (only the numeric rating scale) (31).

#### 2.6.3 Pedagogical staff

The following data and outcomes are collected: days off work due to respiratory tract infections, health-related ability to work using the Work Ability Index (WAI), (32–34); Health Consciousness Scale; health-related quality of life (SF-12); use of TCIM therapists (based on the I-CAM-G); health behaviors/lifestyle habits, and distress (Distress Thermometer, numeric rating scale).

#### 2.6.4 Description of validated outcomes

##### 2.6.4.1 Health consciousness scale-German

The questionnaire originally developed by Gould consists of 9 items in its German version, assessing individual health awareness, on a 5-point Likert scale. These items are grouped into two scales: self-consciousness and self-monitoring. The German version of the HCS was validated by Marsall et al. (22, 23).

##### 2.6.4.2 Short form-12

The SF-12 (version 2) is a shortened version of the Short Form 36 questionnaire (SF-36). It measures the health-related quality of life across 8 domains: “General health perception”, “Physical health”, “Restricted physical-related role function”, “Physical pain”, “Vitality”, “Mental health”, “Restricted emotional-related role function”, “Social functioning”. Those content domains are assigned to two sum scales that represent physical and mental health related quality of life. The 12 questions primarily reference the “past week” and employ different scales with 3–6 gradations along with some “yes/no” questions (24, 25).

##### 2.6.4.3 Modified family climate scale according to Schneewind

The family climate is a significant resource for the mental health of children and adolescents. We used a shortened and modified version of the Family Climate Scale developed by Schneewind et al., which was previously utilized in the KiGGS Survey, to assess family climate (21, 27, 28, 35).

##### 2.6.4.4 Distress thermometer

The NCCN Distress Thermometer developed by the National Comprehensive Cancer Network (NCCN) assesses psychosocial stress in oncology patients using a 0 to 10 scale and a problem list for referrals. A score of 5 or higher indicates that a patient is conspicuously stressed and requires support. Although the distress thermometer was developed for cancer patients, we applied it in this study to healthy individuals because of its simplicity (31).

##### 2.6.4.5 Work ability index

The WAI questionnaire, developed in Finland, assesses individual and group work ability and helps to identify early intervention needs to maintain and promote work fitness. It includes ten questions on physical and mental work demands, health and performance, grouped into seven dimensions. Answers yield a score between 7 and 49 points, with cut-offs for poor (7–27), medium (28–36), good (37–43) and excellent (43–49) work ability (32, 33). This study uses a validated short form of the WAI, in which the 51 individual diseases were replaced with 14 superordinate disease groups (34).

##### 2.6.4.6 Global physical activity questionnaire

The GPAQ developed by the WHO, available in multiple languages, consists of 16 self-administered questions across three domains: occupational, transport-related, and leisure-time physical activity. It measures moderate or vigorous physical activity and sedentary behavior in minutes per day, using metabolic equivalent of tasks (MET) (30, 36, 37). In this study, none of the activity areas were recorded, only the time spent sitting or resting.

#### 2.6.5 Further measurements

Variety and frequency of health-promoting activities, including nutritional offerings in the kindergartens are assessed weekly in both study groups. Parents and staff in the Kneipp group are asked about their views on the KHC under feasibility aspects.

### 2.7 Adverse events

Adverse events (AEs) in children from the Kneipp group are recorded by kindergartens at months 9, 15, and 21 using provided record sheets to log type, onset, duration, intensity, severity and treatment, causal relationship to Kneipp Health Concept, and outcome of each event. With over 500 kindergartens already using Kneipp Health Concept and no serious harms reported, no adverse events are expected. Kindergartens have insurances for all other cases.

### 2.8 Qualitative study part

The qualitative study aims to complement the quantitative data by investigating perceived changes in health awareness, behavior and family health following the introduction of the KHC focusing on a targeted sample of pedagogical staff (educators and kindergarten managers) and parents from the Kneipp group.

The interviews will also identify barriers and facilitators to Kneipp Health Concept implementation in kindergartens, focusing on feasibility, acceptability, and potential improvements. With the interviews we will also explore whether the Kneipp Health Concept extends to families' health behavior and family health, explore its impact on Kneipp skills, and participants' general health awareness. Thirty-two semi-structured interviews were conducted in the Kneipp group at baseline and will be repeated 14–16 months post training, ideally with the same participants to track changes. Interview guides were developed based on a literature review and discussions among the interdisciplinary research team. Baseline interviews were conducted both in person and by telephone, depending on participants' preferences.

To enhance the inter-reliability of the qualitative analysis, a subset of transcripts was independently coded by two researchers (JS and CB). Differences in coding were discussed among the research team until consensus was reached, and the coding framework was subsequently refined accordingly. The codes were then grouped into broader categories by summarizing and synthesizing the coded data, resulting in the development of an analytical framework. This preliminary framework was discussed within the research group involving experts from various disciplines, such as medicine and health service research. It was then applied to the analysis of the remaining interviews.

Triangulation with the quantitative data is planned as part of the mixed methods design. This means that the qualitative data will be used to help understand and interpret the quantitative findings, for example in the domain of lifestyle-related factors, such as individual health consciousness (measured using the Health Consciousness Scale—German, HCS-G), health-related quality of life (assessed via the Short Form-12), and general health behaviors. Similarities and differences between the two datasets will be examined to gain more comprehensive insights and derive further explanations of the quantitative results. Thus, we also aim to minimize errors that may arise from relying on a single data source and will develop a broader and more holistic view of the effects and implementability of the KNEIPP approach. We also aim to enhance the external validity of this research, so that findings may be more likely to be generalized to different contexts.

### 2.9 Sample size

As no confirmatory hypotheses are tested and no primary outcome was defined, no formal sample size was calculated. Sample size was determined by logistical and pragmatical considerations. We planned to include 10 kindergartens (clusters), with around 100 childcare places per site, aiming for at least *n* = 180 children, one parent per child, and *n* = 20 pedagogical staff members in total. These numbers were deemed sufficient for the exploratory assessments of the intervention's effects.

### 2.10 Randomization, matching, and blinding

After enrollment, eligible kindergartens were matched in pairs to improve comparability at the kindergarten (cluster)-level. Kindergartens were matched based on size (40–100 vs. 100–150 children) and location (rural/large vs. town/small town). After matching, randomization was performed within each pair with a 1:1 allocation to the two treatment groups.

The study statistician generated the randomization sequence using SAS 9.4; (SAS Institute Inc. Cary, NC, USA) and informed the Regensburg team after baseline data collection. Kindergarten heads were then notified by telephone and e-mail, and in turn, informed staff, parents, and children about the group allocation.

Since this is an open-label study, participants and study staff are aware of the treatment allocation.

### 2.11 Data collection and management

Participants were able to choose to complete questionnaires and study diaries online or paper based. The type and frequency of Kneipp Health Concept interventions in the observation phase is documented by the kindergarten staff and sent weekly to the Regensburg study center.

Participants receive reminders via phone or emails, if they miss a submission, and those who intend to leave the study are documented on a special study discontinuation form.

After given informed consent, study data is collected and managed using REDCap (Research Electronic Data Capture), a secure, web-based tool (38) hosted at the University of Regensburg. Online responses are directly integrated in the database, while paper-based data is entered by study personnel after quality checks. All data is archived according to GDPR, Good Clinical Practice and recommendations of the Technology and Methods Platform for Networked Medical Research (TMF e. V.). For analyses, data is compiled into a pseudonymized project-specific data set at the Institute of Epidemiology and Preventive Medicine, University of Regensburg and transmitted encrypted to the Institute of Social Medicine, Epidemiology and Health Economics at Charité Universitätsmedizin Berlin for analyses.

During the last 12 months of the assessment period, parents and pedagogical staff are required to report weekly data (e.g., absence days and respiratory tract infections), which, though brief, may be burdensome and result in missing or poor-quality data. Staff shortages and turnover, common in German kindergartens, could also reduce Kneipp Health Concept implementation. To address these issues, we maintain regular contact with the kindergarten management, provide flexible contact offers for parents and staff, and assess potential barriers, throughout the study.

## 3 Anticipated results

We describe as follows the planned analysis of data.

### 3.1 Quantitative data

All quantitative data will be analyzed descriptively. Continuous data will be summarized using means, standard deviations, medians, and quartiles, while categorical data will be presented as frequencies and percentages (overall and by treatment group). Analyses will be conducted at both individual and cluster levels, separately for children, parents and pedagogical staff.

Continuous outcomes will be analyzed using a multilevel model (mixed-effects analysis of covariance) to account for the clustering structure in the data. The model includes treatment group (Kneipp/control) as a fixed effect, cluster (kindergarten) as a random effect, and baseline score (if available) as a covariate. Results will be reported as adjusted means for both treatment groups, 95% confidence intervals and exploratory two-sided *p*-values for group differences. Binary endpoints will be analyzed with multilevel logistic regression. All analyses are exploratory, with no formal significance level or adjustment for multiple testing.

The Full Analysis Set, based on the intention-to-treat principle, will be used for the general analyses, with participants analyzed according to their randomized assignment, regardless of the intervention received. Missing values will generally not be replaced. However, for sensitivity analyses, multiple imputation will be applied for key endpoints, as specified in the statistical analysis plan.

Adherence to treatment and safety data will be analyzed descriptively. All other statistical methods, including sensitivity and subgroup analyses, will be detailed in the statistical analysis plan (SAP) prior to data analysis. An interim analysis is not planned.

### 3.2 Qualitative data

Interviews will be audiotaped, transcribed verbatim, and analyzed with framework analysis using ATLAS.ti (39). This is a systematic approach enabling interdisciplinary research team involvement. The qualitative results will be triangulated with the quantitative findings using a parallel explanative mixed methods approach.

### 3.3 Dissemination policy

The study results will be disseminated through peer-reviewed journals and will also be communicated to the general public in an accessible format. Parents and staff of the kindergartens will get a short summary of the study results in plain language.

Results will be published in peer-reviewed journals and presented at scientific conferences. Authorships will follow author's contributions to the manuscript. Details are described in a publication plan. In addition, individual trial results will be reported to the participants and the general population in lay language.

After publication, data will be available from the corresponding author upon reasonable request, subject to restrictions on scientific purpose and data protection. Access to the final dataset is governed by the cooperation agreement between the two partners, and any use beyond the project period requires written consent from both project partners, respecting third-party rights.

## 4 Discussion

This study will be the first mixed methods, cluster-randomized controlled trial to evaluate the effects of the Kneipp Health Concept in kindergartens in all involved persons: children, parents, and staff. It will provide signals regarding the concept's potential effects in improving health outcomes in this setting. This study will compare the effects of the Kneipp Health Concept in kindergartens by contrasting a group using the Kneipp Health Concept with a control group. The mixed-methods approach offers comprehensive data to better understand the processes and impacts of the Kneipp Health Concept in this setting. Further strength of this study include the study's pragmatic approach that ensures a high level of external validity. The cluster randomization of matched kindergartens based on size and degree of urbanization improves the comparison between groups. The evaluation of data from children, parents and staff allows a comprehensive analysis from different perspectives. However, limited resources in kindergartens due to a lack of time and infrastructure may potentially affect both adherence and data quality. Another limitation may be the exclusive use of self-reported outcomes, which may result in answers influenced by social desirability and recall bias.

Kindergartens in Germany are obliged to use health promoting strategies (nutrition, physical activity) in general, this may potentially resulting in contamination when it comes to the comparison of study groups. To deal with this possible limitation, we collect all health promotion measures implemented in both groups and plan a sensitivity analysis for the frequency of hydrotherapy, taken into account that this is the most unique selling point of the Kneipp Health Concept. Further, the generalizability of data may be limited due to the small number kindergartens and the lack of kindergartens from diverse social and regional backgrounds including the regional focus on Bavaria. Kneipp, as a Bavarian lay-healer and priest is until today more popular in this region than in other parts of Germany. Thus, the acceptance of his methods may vary across Germany.

Another study (DRKS-ID: DRKS00029275) on this topic examines the impact of KHC on children's illness-related absence days in kindergartens (40). Our study extends this by evaluating and comparing various health-related outcomes in children, parents, and pedagogical staff, thus in all those involved in education in the kindergarten setting, both directly and indirectly. In contrast to the other study, we will also conduct follow-up interviews as a part of the qualitative study, to gain insights into the feasibility and challenges of the Kneipp Health Concept in the course of time and to explore its long-term impact.

In conclusion, the Kneipp Health Concept will be scientifically evaluated and if necessary, optimized after evaluation of the results of our study. This will pave the way for a sustainable implementation of the Kneipp Health Concept. In addition, this exploratory study will inform the planning of future intervention studies with valuable data material and contribute to the development and evaluation of effective childhood prevention strategies.

## Author's note

The reporting of the study follows the SPIRIT Checklist 2022 for protocol publications (Supplementary material 2).

## Data Availability

The original contributions presented in the study are included in the article/Supplementary material, further inquiries can be directed to the corresponding author.
