# Supplementary material for: Health promotion in German kindergartens—design and methods of the exploratory, cluster-randomized, mixed methods KNEIPP–KITA Bavaria study
Source: Front Med (Lausanne). 2025 May 29;12:1585322. doi: 10.3389/fmed.2025.1585322 (PMC12160532; doi:10.3389/fmed.2025.1585322)
Supplement: Supplementary file 1 [file Supplementary_file_2.docx]

Supplement Table 2: *Data assessment time points*

| **Assessement**  **Questionnaires/*weekly journals*** | -3 months before training (baseline)  T1 | 9 months after training  T2 | 15 months after training  T3 | 21 months after training  T4 |
| --- | --- | --- | --- | --- |
| **Children (assessment by parents)** |  |  |  |  |
| Age | X |  |  |  |
| Sex | X |  |  |  |
| Height and weight | X | X | X | X |
| Center of life, number of siblings | X | X | X | X |
| Health status: general/allergies/chronic diseases | X | X | X | X |
| Number of URTI last 6 months | X | X | X | X |
| Days with URTI last 3 months | X | X | X | X |
| Absence days in the kindergarten during the last 3 months | X | X | X | X |
| Hospitalized due to URTI last 12 (baseline) /6 months | X | X | X | X |
| Quality of life (KINDL-R questions) | X | X | X | X |
| Wellbeing of the child in the kindergarten  Since when in the kindergarten?  How many hours of daily care?  Lunch in the kindergarten? | X  X  X  X | X  X  X | X  X  X | X  X  X |
| Lifestyle: Physical activity (frequency last 4 weeks)/ Screentime (h/week) last week / nutrition last 4 weeks | X | X | X | X |
| Use of physician/ therapists last 6 months | X | X | X | X |
| *URTI weekly journal: number of URTI days, symptoms, severity, treatment, absence days (continuously T2-T4)* |  | X | X | X |
| *Number of absence days (kindergarten) in general (from regular kindergarten documentation, continuously T1-T4)* | X | X | X | X |
| **Parents** |  |  |  |  |
| Who is answering the questionnaire? | X | X | X | X |
| Migration background | X |  |  |  |
| Age, sex, legal status | X |  |  |  |
| Education: highest educational level, highest vocational training, profession | X |  |  |  |
| Health consciousness (HCS) | X | X | X | X |
| Health related quality of life (SF-12) | X |  | X | X |
| Distress Thermometer | X | X | X | X |
| Use of Integrative Medicine therapists during the last 12 months | X |  |  | X |
| Family climate | X |  |  | X |
| Lifestyle: Physical activity, nutrition, smoking habits | X |  |  | X |
| Kneipp elements used  - type, frequency  - effects on health status  - known since when? | X |  |  | X |
| Kneipp group only:  - Perceived effects for health promotion in the family  - Satisfaction with KHC, evaluation |  | X  X |  | X  X |
| **Pedagogical staff** |  |  |  |  |
| Age | X |  |  |  |
| profession | X |  |  | X |
| Migration background | X |  |  |  |
| Health consciousness (HCS) | X | X | X | X |
| Use of Integrative Medicine therapists during the last 12 months | X |  |  | X |
| Work ability last 12 months (WAI) | X |  |  | X |
| Health related quality of life (SF-12) | X |  | X | X |
| Distress Thermometer | X | X | X | X |
| Lifestyle: Physical activity, nutrition, smoking habits | X |  |  | X |
| Number of days with URTI last 3 months | X | X | X | X |
| Days absent from work due to URTI last 3 months | X | X | X | X |
| Kneipp elements used  - type, frequency  - Perceived effects on health status  - known since when? | X |  |  | X |
| Kneipp group only:  -improvement in relationship to children/team  -Implementable in daily work?  - effects on health consciousness/health  - satisfaction with KHC |  | X | X | X |
| *URTI weekly journal: number of days, symptoms, severity, treatment, absence days (continously T2-4)* |  | X | X | X |
| **Kindergartens** |  |  |  |  |
| Structural features: Size/provider/location/staffing ratio/number of groups and group size  Total number of pedagogical staff, facilities (outdoor areas, number of group rooms, additional rooms for physical activity, music, workshop, etc.) | X  X | X | X | X |
| Type of offers/specification in the last 4 weeks in the areas of nutrition/physical activity/relaxation/water applications/herbs | X | X | X | X |
| Adverse events (Kneipp group only) |  | X | X | X |
| *Weekly journal:*  *Number of offers in the areas of nutrition/physical activity /relaxation/water applications/herbs per week, Outdoortime per week (T2-T4)* |  | X | X | X |

URTI: upper respiratory tract infection, mo= month, T= timepoint, h=hour; HCS=Health consciousness scale, WAI = work ability index, SF- 12 = Short form 12
